# Statins and New-Onset Diabetes Mellitus: LDL Receptor May Provide a Key Link

**DOI:** 10.3389/fphar.2017.00372

**Published:** 2017-06-13

**Authors:** Qi Yu, Ying Chen, Cang-Bao Xu

**Affiliations:** ^1^Institute of Basic and Translational Medicine, Xi’an Medical UniversityXi’an, China; ^2^Shaanxi Key Laboratory of Ischemic Cardiovascular DiseaseXi’an, China; ^3^Institute of Material Medical, School of Pharmacy, The Fourth Military Medical UniversityXi’an, China; ^4^Department of Information and Communication Engineering, Xi’an Jiaotong UniversityXi’an, China

**Keywords:** type 2 diabetes mellitus, statin, low-density lipoprotein receptor, familial hypercholesterolemia, islet

## Abstract

Numerous studies have noted that populations treated with statins have increased risk for new-onset diabetes mellitus; however, the underlying molecular mechanisms are not fully understood. Interestingly, familial hypercholesterolemia (FH) patients with mutations in the low-density lipoprotein receptor (LDLR) gene are protected against diabetes mellitus (DM), despite these patients being subjected to long-term statin therapy. Since the common pathway between FH and statin therapy is LDLR-mediated cellular cholesterol uptake, the arising question is whether the LDLR plays an important role in the diabetogenic effect of statins. Indeed, given that statins can regulate the LDLR expression in liver and peripheral tissue, there is a possible mechanism that the increased LDLR causes cellular cholesterol accumulation and dysfunction in pancreatic islets, explaining why statins fail to increase the risk of DM in FH patients. In this paper, with regarded to recent literatures, we highlight the role of LDLR in the pathophysiology of cholesterol-induced pancreatic islets dysfunction, which may provide the key link between statins treatment and the increased risk of new-onset diabetes mellitus.

## Introduction

Statins are first-line drugs for treating hypercholesterolemia and used for primary and secondary CVD prevention. However, with the increasing clinical application of statins, accumulated evidence from clinical trials suggests that statins can increase the risk of new-onset DM, but the underlying pathological mechanism remains to be determined (Brault et al., 2014). Statins are 3-hydroxy-3-methyl-glutaryl coenzyme-A reductase (HMG-CoA reductase) inhibitors, which were first approved for clinical use by the FDA in 1987. In addition to inhibiting cellular cholesterol synthesis, statins also up-regulate the LDLR in the liver and peripheral tissues, resulting in increased blood LDL-C removal and reduced CVD risk (Beltowski et al., 2009). However, the following great mystery remains: why do statins reduce blood cholesterol but also cause T2DM? After all, hypercholesterolemia is also an important risk factor for T2DM.

Previous studies suggest that statins also act by inhibiting glucose transporter 4, isoprenoid, Coenzyme Q10, Dolichol Biosynthesis and other mechanisms to cause insulin resistance and diminished insulin secretion in patients (Beltowski et al., 2009; Axsom et al., 2013; Brault et al., 2014). Nevertheless, these findings usually depend on the investigation of individual statins, and the mechanism underlying the general diabetogenic effect of statins is unknown. More importantly, these studies do not address whether cholesterol metabolism is involved with the diabetogenic effect of statins, even though the main pharmacological effect of statins is to decrease blood cholesterol.

Fortunately, a serial of studies based on a population with FH have shed light on these questions (Yu et al., 2016). FH patients were reported to present a relatively low incidence of DM in contrast to their unaffected relatives or hyperlipidemic patients, even when these patients were subjected to intensive statin treatment (Besseling et al., 2015). Since most FH patients in these studies have heterozygous mutation in the LDLR gene, an interesting question arises as to whether LDLR mediated cellular cholesterol metabolism is involved with the diabetogenic effect of statins (Preiss and Sattar, 2015; Besseling and Hutten, 2016). Indeed, dysregulation of cellular cholesterol metabolism severely impairs the function of pancreatic β cells, as has been shown by *in vitro* and *in vivo* studies (Brunham et al., 2008). Moreover, in a mouse model lacking the LDLR, the pancreatic β cells are protected from accumulation of cholesterol and cholesterol-induced β cell dysfunction, whereas mice carrying more LDLR exhibit pancreatic islet abnormalities (Kruit et al., 2010; Mbikay et al., 2010). Overall, these novel findings lead us to speculate that LDLR-mediated cellular cholesterol metabolism may associate with the diabetogenic effect of statins.

## Association between Stains Intake and the Risk of New-Onset T2DM

The association between statin treatment and the prevalence of T2DM has been examined extensively. This association was first reported by Ridker et al. (2008), with their data suggesting that rosuvastatin had a higher incidence of physician-reported DM. A meta-analysis with 91140 participants covering 13 statins showed that statin therapy was associated with a 9% increased risk for incident DM (Sattar et al., 2010). In the SPARCL trial, a population with statin treatment had an 11% higher risk of DM than the placebo group. Moreover, Culver et al. reported a 48% increase in the risk of new-onset DM among women prescribed a statin, after adjusting for potential confounding factors (Culver et al., 2012). Similarly, a population-based study showed that treatment with higher atorvastatin, simvastatin or rosuvastatin doses was associated with an increased risk for new-onset DM (22, 10, and 18%, respectively) (Carter et al., 2013). A recent study concluded that statin users were more commonly diagnosed with DM and diabetic complications even after adjustment for potential confounding factors during the follow-up period (Mansi et al., 2016). In regards to these numerous clinical trials and meta-analyses, the data strongly support statins modestly increasing the risk for DM (Sattar and Taskinen, 2012). Nevertheless, it is important to note that statins are highly effective for prevention of cardiovascular events in individuals with or without DM (Axsom et al., 2013). The Cholesterol Treatment Trialist investigators concluded that the net absolute benefit observed with statin therapy in such individuals is more than 50 times larger than any putative effect on DM (Hennekens et al., 2017). As a result of clinical observations, numerous studies suggest that statins are irreplaceable for the prevention and treatment of CVD (Axsom et al., 2013). In view of the fact that statins have a very important role in the primary and secondary prevention of CVD, the underlying mechanism that are responsible for the diabetogenic effect of statins and novel strategies for reducing the side effects should therefore be explored.

## Inverse Association of FH and the Risk of T2DM

To our knowledge, the low risk of DM in FH patients was first reported in 1997 by Vohl et al. (1997). They found that the prevalence of DM was higher in the non-FH group, reaching 18.7% compared with 3.9% in the FH patients with a defective allele at LDLR and 7.9% in FH patients with a null mutation at LDLR (**Table 1**) (Vohl et al., 1997). This phenomenon was confirmed by another cross-sectional study, showing that familial combined hyperlipidaemia (FCH) patients had a significantly increased prevalence of DM (13%) vs an FH group with LDLR mutations (2%) (**Table 1**) (Skoumas et al., 2007). Skoumas et al. (2014) presented a study regarding the role of long duration statin treatment on DM incidence of FH patients with LDLR mutations, reporting that 14% of FCH and only 1% of FH patients developed DM during follow-up (**Table 1**). They also concluded that long duration of high statin treatment did not seem to be associated with diabetic risk in FH patients. These results were confirmed by other studies (**Table 1**) (Kusters et al., 2014; Fuentes et al., 2015). A recent cross-sectional study may provide some novel insights into the inverse association between FH and prevalence of DM (**Table 1**) (Besseling et al., 2015). Data from this study suggested that the prevalence of T2DM in FH patients was 50% lower than that in unaffected relatives despite these FH patients showing greater statins use. Interestingly, they observed an inverse relationship between the severity of the FH causing mutation and the prevalence of T2DM, indicating that patients with LDLR-negative mutations have the lowest prevalence of T2DM. Taking these results together, a novel phenomenon is raised, whereby FH patients with LDLR mutations may be protected against T2DM and the diabetogenic effect of statins.

**Table 1:** Summary of population studies investigating onset of DM in FH patients with statins treatment.

Author and Published year	Country	Study design	Population and the cause of FH	DM-related findings
Vohl et al., 1997	Canada	Case control study	102 patients without FH, 102 hFH patients; a defective allele at LDLR or LDLR mutation.	The prevalence of DM was significantly higher in the non-FH group than in the two FH groups (*P* < 0.05).
Skoumas et al., 2007	Greece	Cross-sectional study.	A total 1306 subjects: 600 individuals with hFH, and 706 individuals with FCH; LDLR mutation or plasma levels of LDL cholesterol above the 95th percentile.	FCH had a significantly increased prevalence of DM (13 vs. 2%, *P* < 0.001) vs FH group, whereas total cholesterol, LDL-cholesterol, and apolipoprotein B levels were higher (all *P* < 0.001) in FH subjects.
Skoumas et al., 2014	Greece	Ambispective cohort study.	A total of 523 adult patients (314 hFH and 209 FCH patients); LDL-receptor mutation or plasma levels of LDL cholesterol above the 95th percentile.	14% of FCH and only 1% of hFH patients developed DM during follow up.
Kusters et al., 2014	Netherland	Retrospective cohort study	2144 children with hFH; LDR mutation.	Statin treatment was not associated with an increased risk of new-onset DM in these patients.
Besseling et al., 2015	Netherland	Cross-sectional study	All individuals (*n* = 63 320) who underwent DNA testing for FH; 3475 were ApoB mutation carriers, 21 606 had the LDLR mutation, and 56 had PCSK9 mutation.	The prevalence of T2DM was 1.75% in FH patients (*n* = 440/25 137) vs 2.93% in unaffected relatives (*P* < 0.001). The adjusted prevalence of type 2 DM by APOB vs LDL receptor gene was 1.91% vs 1.33%.
Fuentes et al., 2015	Spain	Cross-sectional and prospective cohort study	2558 FH and 1265 unaffected relatives with a mean follow-up of 5.9 years; LDLR mutation.	Finally, in the adjusted Kaplan–Meier curve, there are no differences between FH group vs control group in the incidence of T2DM according the duration of treatment with statins.

*hFH, heterozygous Familial Hypercholesterolemia; FCH, familial combined hyperlipidemia; LDLR, low density lipoprotein receptor; T2DM, type 2 diabetes mellitus; PCSK9, proprotein convertase subtilisin/kexin type 9; ApoB, apolipoprotein B*.

## Regulation of LDLR Expression by Statins

**FIGURE 1:**
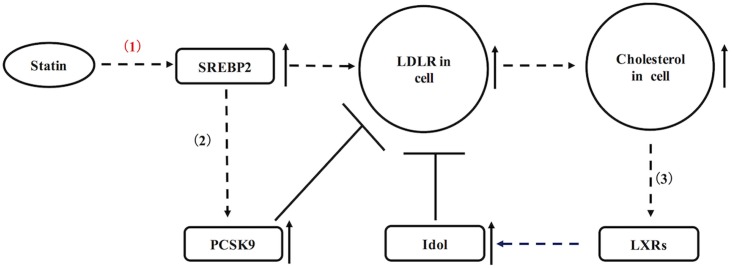
A schematic diagram representing the regulation of hepatic LDLR by statins. (1) Statins up-regulate LDLR via SREBP2. (2) SREBP2 also increases transcription of PCSK9 to degrade LDLR. (3) Cellular cholesterol accumulation may activate LXR, which up-regulates Idol to degrade LDLR.

As mentioned above, there are two ways of that statins reduce the blood cholesterol: by inhibited HMG-CoA reductase, statins can efficiently reduce formation of LDL-C in blood; additionally, statins also up-regulate LDL receptor in liver and peripheral tissues, resulting in increased blood LDL-C removal (Beltowski et al., 2009). Of a pathway in the regulation of LDLR, statins can cause up-regulation of sterol regulatory element-binding protein 2 (SREBP-2) in hepatocytes (**Figure 1**). SREBP-2 is known as a transcription factor, which efficiently stimulates transcription of LDLR and other sterol-regulated genes (**Figure 1**) (Brown and Goldstein, 1997). As a result of statin incubation, LDLR and its mRNA levels are raised in Hep G2, human and rat hepatocytes (Qin et al., 1992). In statin-treated dogs, they usually show increased hepatic LDLR as previously reported (Alberts et al., 1980). Nevertheless, LDLR expression in statins-treated animals also is not extremely high, because LDLR is subjected to negative feedback regulations as well (**Figure 1**). On the one hand, cellular cholesterol accumulation may lead to increased oxysterols, which are the natural ligands for liver X receptor (LXR). Activation of LXR can positively regulate transcription of inducible degrader of the LDLR (Idol, a ubiquitin ligase) to degrade the LDLR (**Figure 1**) (Hong et al., 2014). On the other hand, SREBP-2 also up-regulates PCSK9 in liver, which is a powerful enzyme to mediate the degradation of LDLR (**Figure 1**) (Raal et al., 2015). In contrast to liver, the regulation of LDLR in pancreatic islets has not been fully elucidated. If LDLR expression in pancreatic islets is subjected to similar regulations as shown in liver, there is an interesting question whether negative feedback regulation is affected in statins-treated patients with the pathological condition.

## Lipid-Lowering Therapy, LDLR and Dysfunction of Pancreatic Islets

Hyperlipidaemia increases the risk for both CVD and T2DM, suggesting that lipid-lowering drugs for treating CVD may also have protective effects on pancreatic islets and thus prevent the onset of T2DM. For instance, bezafibrate that is a fibrate drug used as a lipid-lowering agent to treat hyperlipidaemia, is also proven that can reduce the incidence and delay the onset of T2DM in high risk patients (Tenenbaum and Fisman, 2012). In addition, patients received ezetimibe and colesevelam for treating dyslipidaemia might also be shown to decrease the risk of the onset of T2DM (Athyros et al., 2014). In animal model, statins had some beneficial effects on pancreatic islets, e.g., atorvastatin preserved pancreatic β cell function in obese C57BL/6 J mice (Chen et al., 2014), and pitavastatin suppressed high fat diet (HFD)-induced the pathogenesis of pancreatic islets in rats (Mizukami et al., 2012). However, it is well known that statins lower LDL cholesterol, while they have negative effects on pancreatic islets. The molecular mechanisms behind this side effect involve with an increased LDLR-mediated uptake of LDL-C in islets via statins-induced up-regulation of the LDLR expression. Most likely, there is a potential mechanism that the pancreatic islets are impaired by abnormal cholesterol levels via the up-regulation of LDLR expression and subsequent increased LDLR-mediated uptake of LDL-C. Thus, LDLR mutation in patients with FH may prevent the onset of T2DM as well as the diabetogenic effect of statins. Indeed, numerous studies have revealed that the accumulation of cholesterol in the pancreatic islets leads to dysfunction of pancreatic β cell (Brunham et al., 2008; Fryirs et al., 2009). *In vitro*, the addition of LDL-C to rat islet β cells results in cell death in an LDLR-depended manner (Roehrich et al., 2003), while the inhibitory effect of LDL on insulin secretion can be abolished by LDLR deficiency (Rutti et al., 2009). Furthermore, increased LDLR may allow more modified LDL-C entering islet β cells and finally causes cytotoxicity to these cells. *In-vivo* studies provide further evidences that the LDLR may play a key role in islet function (Sattar and Taskinen, 2012). Using the LDLR-deficient mouse, Kruit et al. (2010) have found that lack of LDLR protects pancreatic β cells from the accumulation of cholesterol and cholesterol-induced β cell dysfunction. Furthermore, PCSK9-null male mice with more LDLR in pancreatic islet β cells exhibit impaired glucose tolerance and pancreatic islet abnormalities (Mbikay et al., 2010). Similarly, variations in PCSK9 and HMGCR are associated with nearly identical protective effects on the risk of cardiovascular events but are also associated with very similar effects on the risk of DM (Ference et al., 2016). Not only the above two genes, LDL-C-lowering genetic variants in a number of genes are found that are associated with a higher risk of T2DM, which may further confirm the potential adverse effects of LDL-C-lowering therapy (Lotta et al., 2016). Combined with the FH studies, these results strongly support a model whereby LDLR-mediated uptake of LDL-C and cellular cholesterol accumulation is the pathological basis for the prevalence of T2DM and the diabetogenic effects of statins.

## Conclusion

The common pathway in FH and statin-induced DM is LDLR-mediated uptake of LDL-C, and elucidation of this pathway may help us to understand the potential mechanism for the diabetogenic effects of statins. Moreover, this pathway closely associates with the pharmacological effect of statins but is independent of the type of statin. If this pathway is proven, we can not only utilize it to prevent the diabetogenic effects of statins but can also target this pathway to treat T2DM.

## Author Contributions

QY proposed this hypothesis and analyzed the literatures and wrote the manuscript. YC collected the literatures and revised the manuscript. CBX contributed to crucial revisions in the manuscript.

## Conflict of Interest Statement

The authors declare that the research was conducted in the absence of any commercial or financial relationships that could be construed as a potential conflict of interest.

## Abbreviations

CVD: cardiovascular disease
DM: diabetes mellitus
FCH: familial combined hyperlipidemia
FH: familial hypercholesterolemia
LDL-C: low density lipoprotein-cholesterol
LDLR: low-density lipoprotein receptor
PCSK9: proprotein convertase subtilisin/kexin type 9
T2DM: type 2 diabetes mellitus.
